# Managing thrombotic risk in patients with diabetes

**DOI:** 10.1186/s12933-022-01581-x

**Published:** 2022-08-22

**Authors:** A. John Camm, Hani Sabbour, Oliver Schnell, Francesco Summaria, Atul Verma

**Affiliations:** 1 Division of Cardiac and Vascular Sciences, Molecular and Clinical Sciences Research Institute, St George’s, University of London, London, Cranmer Terrace, SW17 0RE UK; 2 Heart and Vascular Institute, Cleveland Clinic Abu Dhabi, Abu Dhabi, United Arab Emirates; 3 Warren Alpert School of Medicine, Brown University, Rhode Island, USA; 4 Forschergruppe Diabetes e.V., Neuherberg, Munich, Germany; 5 Department of Cardiology, San Eugenio Hospital, Rome, Italy; 6 Southlake Regional Health Centre, Newmarket, ON Canada; 7 Department of Medicine, University of Toronto, Toronto, ON Canada

**Keywords:** Atrial fibrillation, Diabetes mellitus, NOAC, Non-vitamin K antagonist oral anticoagulants, Thrombosis

## Abstract

It is well known that diabetes is a prominent risk factor for cardiovascular (CV) events. The level of CV risk depends on the type and duration of diabetes, age and additional co-morbidities. Diabetes is an independent risk factor for atrial fibrillation (AF) and is frequently observed in patients with AF, which further increases their risk of stroke associated with this cardiac arrhythmia. Nearly one third of patients with diabetes globally have CV disease (CVD). Additionally, co-morbid AF and coronary artery disease are more frequently observed in patients with diabetes than the general population, further increasing the already high CV risk of these patients. To protect against thromboembolic events in patients with diabetes and AF or established CVD, guidelines recommend optimal CV risk factor control, including oral anticoagulation treatment. However, patients with diabetes exist in a prothrombotic and inflammatory state. Greater clinical benefit may therefore be seen with the use of stronger antithrombotic agents or innovative drug combinations in high-risk patients with diabetes, such as those who have concomitant AF or established CVD. In this review, we discuss CV risk management strategies in patients with diabetes and concomitant vascular disease, stroke prevention regimens in patients with diabetes and AF and how worsening renal function in these patients may complicate these approaches. Accumulating evidence from clinical trials and real-world evidence show a benefit to the administration of non-vitamin K antagonist oral anticoagulants for stroke prevention in patients with diabetes and AF.

## Introduction

Diabetes is a well-established cardiovascular (CV) risk factor, nearly doubling the risk of vascular outcomes, such as coronary heart disease, ischaemic stroke and vascular death [1]. People with diabetes are also at risk of major adverse limb events (MALE), with up to 13% of the global population of diabetes experiencing diabetic foot and limb complications as a result of peripheral vascular disease in the lower limbs or neurological disorders [2]. CV disease (CVD) is a leading cause of mortality and morbidity in patients with type 2 diabetes, accounting for approximately 50% of deaths in the patient population [3]. The CV risk in patients with diabetes can be identified based on various characteristics of the disease, including the duration of the disease, the age of the patient, the type of diabetes and the presence of additional risk factors [4]. These risk factors include a high body mass index, hypertension, dyslipidaemia, smoking, a family history of premature coronary disease and chronic kidney disease (CKD) (Table 1) [4–8].

**Table 1:** ESC guidelines on cardiovascular risk categories in patients with diabetes [4, 7, 8]

Category	Guidelines on diabetes, pre-diabetes and cardiovascular diseases [4]	Guidelines for the management of chronic coronary syndromes [7]	Guidelines for the management of dyslipidaemias in collaboration with the EAS [8]
Very high risk	Concomitant established cardiovascular disease		Target organ damage^c^
	Other target organ damage^a^		≥ 3 major risk factors
	≥ 3 major risk factors^b^		Early onset T1DM for > 20 years
	Early onset T1DM for > 20 years		
High risk	Diabetes ≥ 10 years without target organ damage and an additional risk factor	Diffuse multivessel CAD	Diabetes ≥ 10 years without target organ damage and an additional risk factor
Moderate risk	Young patients^d^ with diabetes for < 10 years without an additional risk factor	Any of recurrent MI, PAD, HF, or CKD with eGFR 15–59 mL/min/1.73 m^2^	Young patients^d^ with diabetes for < 10 years without an additional risk factor

CAD, coronary artery disease; CKD, chronic kidney disease; EAS, European Atherosclerosis Society; ESC, European Society of Cardiology; eGFR, estimated glomerular filtration rate; HF, heart failure; MI, myocardial infarction; PAD, peripheral artery disease; T1DM, type 1 diabetes mellitus; T2DM, type 2 diabetes mellitus

^a^Renal impairment (eGFR ≥ 30 mL/min/1.73 m^2^), left ventricular hypertrophy, proteinuria or retinopathy

^b^Age, dyslipidaemia, hypertension, obesity or smoking

^c^Microalbuminuria, retinopathy or neuropathy

^d^T1DM aged < 35 years or T2DM aged < 50 years

**Fig. 1:**
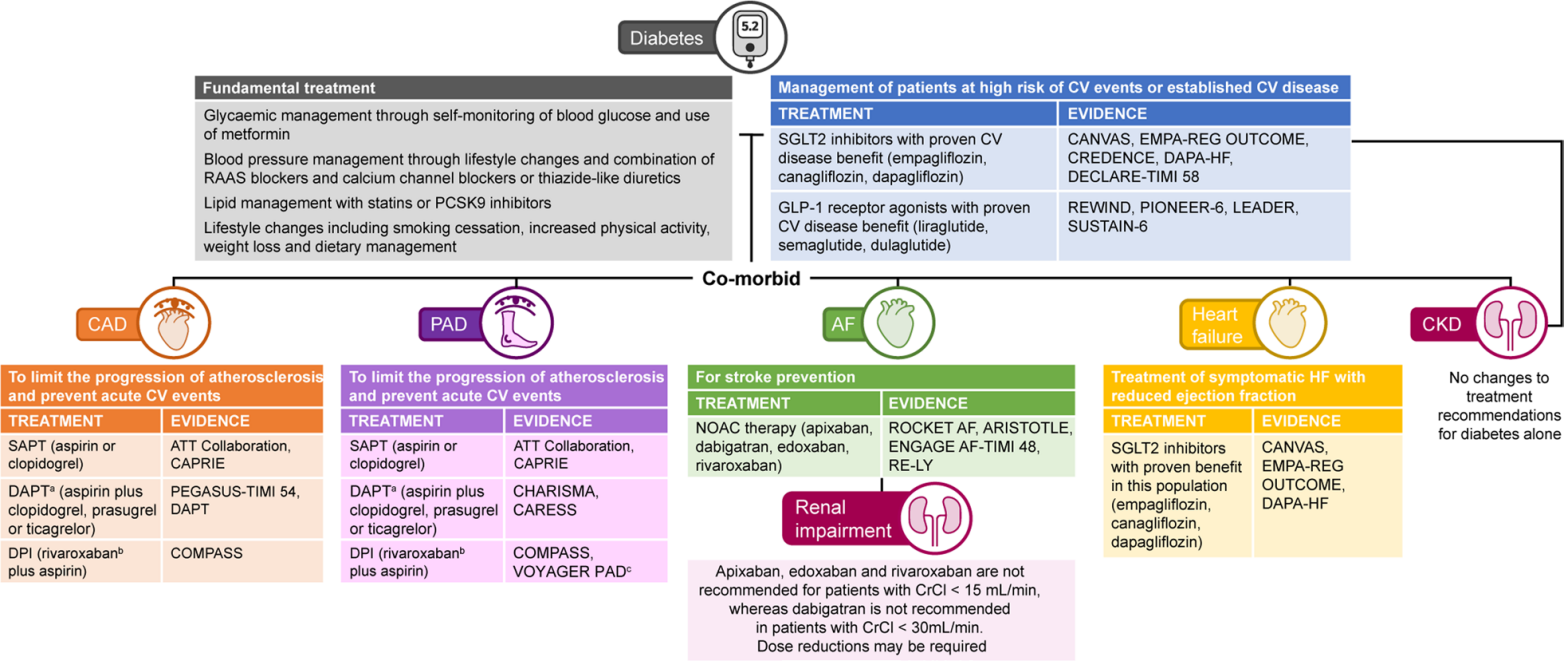
Management of thrombotic risk in patients with diabetes and co-morbidities. All studies listed included patients with diabetes. ^a^Only recommended for patients with prior MI who have tolerated DAPT for 1 year. ^b^Rivaroxaban is contraindicated in patients with CrCl < 15 mL/min. ^c^Post-peripheral revascularization. Published after the latest guideline recommendation. Guidelines recommend rivaroxaban 2.5 mg bid plus aspirin for patients with symptomatic PAD at high risk of ischaemic events. AF, atrial fibrillation; ATT, Antithrombotic Triallists’ Collaboration; bid, twice daily; CAD, coronary artery disease; CKD, chronic kidney disease; CrCl, creatinine clearance; CV, cardiovascular; DAPT, dual antiplatelet therapy; DPI, dual pathway inhibition; GLP-1, glucagon-like peptide-1; NOAC, non-vitamin K antagonist oral anticoagulant; PAD, peripheral artery disease; PCSK9, proprotein convertase subtilisin/kexin type 9; RAAS, renin–angiotensin–aldosterone system; SAPT, single antiplatelet therapy; SGLT2, sodium-glucose co-transporter-2

The primary treatment of patients with diabetes to prevent CV events includes lifestyle changes such as weight loss, smoking cessation and dietary management, along with the monitoring of glycaemic, blood pressure and lipid levels (Fig. 1). The use of an antiplatelet agent for the primary prevention of CV events in patients with diabetes has largely focused on aspirin and has recently been reviewed [9]. A 2009 meta-analysis of six primary prevention trials conducted by the Antithrombotic Trialists’ Collaboration found that aspirin significantly reduced serious CV events [myocardial infarction (MI), stroke or CV death] by 12% per year compared with controls in patients with diabetes at low risk of CVD, but at a cost of significantly increased gastrointestinal (GI) and extracranial bleeding events [10]. In contrast, in the Clopidogrel for High Atherothrombotic Risk and Ischemic Stabilization, Management, and Avoidance (CHARISMA) trial involving a subgroup of patients with multiple atherothrombotic risk factors (≈80% of whom had diabetes), an increase in serious CV events (MI, stroke or CV death, including haemorrhage) was observed in patients receiving clopidogrel plus aspirin versus those receiving aspirin alone (6.6% vs 5.5%, *P* = 0.20). However, increased rates of Global Use of Strategies to Open Occluded Coronary Arteries (GUSTO)-defined severe and moderate bleeding were observed in patients in the subgroup receiving clopidogrel plus aspirin compared with those receiving aspirin alone (2.0% vs 1.2%, *P* = 0.07 and 2.1% vs 1.3%, *P* < 0.001 for severe and moderate bleeding, respectively) [11].

More recently, the A Study of Cardiovascular Events in Diabetes (ASCEND) trial also demonstrated a significant reduction in the rate of serious CV events (composite of non-fatal MI or stroke, transient ischaemic attack or CV death, excluding intracranial haemorrhage) over a mean follow-up of 7.4 years in patients with diabetes and no evidence of CVD with aspirin compared with placebo [8.5% vs 9.6%, rate ratio = 0.88, 95% confidence interval (CI) 0.79–0.97; *P* = 0.01]. Similarly, this benefit was offset by an increase in major bleeding, mainly attributed to GI bleeding (4.1% vs 3.2%, rate ratio = 1.29, 95% CI 1.09–1.52; *P* = 0.003) [12]. In the Aspirin in Reducing Events in the Elderly (ASPREE) trial of community-dwelling older adults, of whom 11% had diabetes, there was no reduction in the composite CVD endpoint (stroke, MI, fatal coronary heart disease or hospitalization for heart failure [HF]) with aspirin compared with placebo; however, a significantly increased risk of major bleeding was observed [13]. In the Aspirin in Primary Prevention of cardiovascular disease in diabetes (APPRAISE) meta-analysis of 12 trials of patients with diabetes and no history of CVD that compared the use of aspirin versus placebo, including ASCEND and ASPREE, a reduction in major adverse CV events (MACE) was found. No significant difference in bleeding events with aspirin use was found in this meta-analysis, although it should be noted that this conclusion may be imprecise owing to the wide CIs associated with the estimates [14]. However, a Japanese trial of 2539 patients with type 2 diabetes who did not have CVD did not find a reduction in CV events with aspirin compared with no aspirin [hazard ratio (HR) = 1.14, 95% CI 0.91–1.42; *P* = 0.2]. Nevertheless, an increase in GI bleeding was observed in patients receiving aspirin compared with those not receiving aspirin (2% vs 0.9%, *P* = 0.03) [15]. As a result of these studies, guidelines recommend that after an assessment of bleeding risk, antiplatelet therapy with aspirin may be considered for the primary prevention of CVD in patients with diabetes at a high risk of CV events but is not recommended in patients at moderate or low risk of CV events [4, 5].

Approximately 32% of patients with type 2 diabetes worldwide have existing CVD [3]. Patients with diabetes and established CVD, such as coronary artery disease (CAD) or peripheral artery disease (PAD), have a particularly elevated risk of CV events [4, 16]. An analysis of the REduction of Atherothrombosis for Continued Health (REACH) registry revealed that the 4-year risk of CV death, MI or stroke was 20% in patients with stable atherosclerosis and diabetes [16]. However, patients remain at risk despite traditional CV risk reduction approaches of CV risk factor control and antiplatelet therapy, with approximately 50% of deaths in patients with diabetes being attributed to CVD [3].

**Fig. 2:**
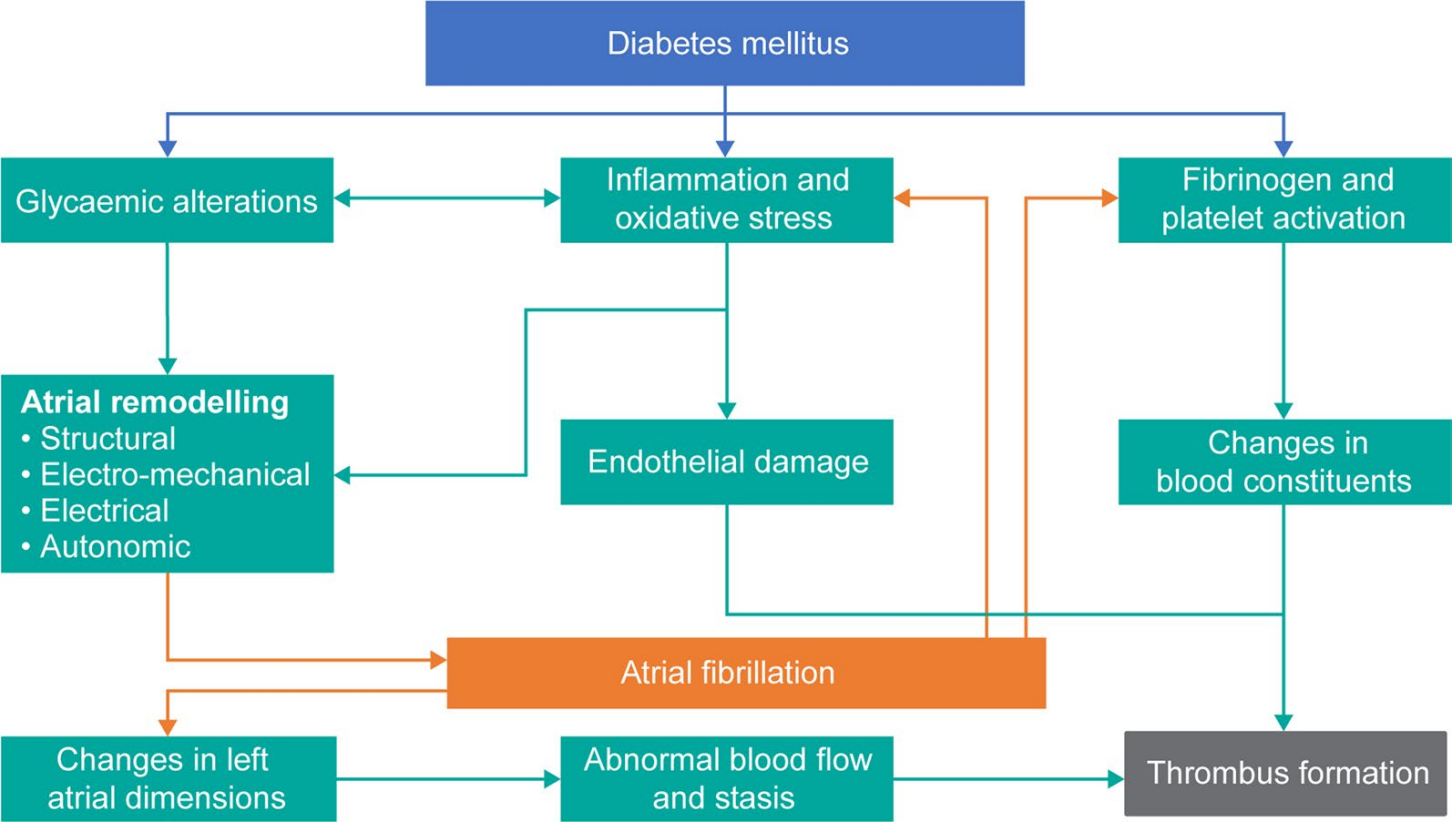
Pathophysiology of the link between atrial fibrillation and diabetes [24, 25]

Diabetes is also prevalent in patients with atrial fibrillation (AF), a common sustained cardiac arrhythmia associated with an increased risk of stroke [20–22]. Diabetes, an independent risk factor for AF itself [23], has been shown to increase the risk of stroke and mortality in patients with AF by several mechanisms (Fig. 2) [20, 22, 24, 25].

The coexistence of AF and CAD, which raises therapeutic and safety concerns, is more frequently observed in diabetic populations compared with the general population [20]. Recent studies have provided conflicting evidence of the effectiveness of sodium-glucose co-transporter-2 (SGLT2) inhibitors, dipeptidyl peptidase-4 inhibitors and glucagon-like peptide-1 (GLP-1) receptor agonists in demonstrating a reduction in incident AF in patients with diabetes [26–29]. In patients with AF and additional risk factors, such as diabetes, oral anticoagulant therapy is the cornerstone of treatment for the prevention of thromboembolic events [4, 30, 31].

Patients with diabetes exist in a prothrombotic and inflammatory state as a result of endothelial dysfunction and platelet hyperactivity, and a reduction in clot dissolution [25]. Therefore, more potent antithrombotic agents or novel drug combinations may provide greater clinical benefit for high-risk patients with diabetes, such as those who also have AF or established CVD. This review will discuss current and future management approaches to improve CV outcomes in these high-risk patient populations. Figure 1 provides an overview of these approaches, and the supporting evidence, for patients with diabetes and co-morbid CAD, PAD, HF, CKD, AF and concomitant renal impairment.

## CV risk management strategies in patients with diabetes and CAD and/or PAD

Current guidelines for the management of CV risk in patients with diabetes and CAD and/or PAD recommend CV risk factor control to limit the progression of atherosclerosis and antithrombotic therapy to prevent acute CV events (Table 1) [4, 5, 7, 17–19]. The control of CV risk factors in patients with diabetes involves a multifactorial approach including lifestyle modifications, as well as glucose, lipid and hypertension control [5].

The standard of care for the secondary prevention of acute CV events in patients with CAD and prior MI or revascularization, and in patients with symptomatic PAD is single antiplatelet therapy with aspirin or clopidogrel [4, 5, 7, 17]. Despite the use of single antiplatelet therapy, these patients remain at high risk of CV and MALE [32]. According to Dick et al*.* 2007, nearly half of patients with critical limb ischaemia had diabetes [32]. Furthermore, patients with diabetes and PAD are at a five times greater risk of amputation and three times greater risk of mortality than patients without diabetes [33]. In a sub analysis of patients with diabetes in the Clopidogrel Versus Aspirin in Patients at Risk of Ischaemic Events (CAPRIE) trial, the incidence of ischaemic events was reduced with clopidogrel compared with aspirin; however, the rate was still high; event rates were 15.6% and 17.7% per year for patients with diabetes treated with clopidogrel and aspirin, respectively [34].

Various trials have investigated the efficacy and safety of intensified antiplatelet therapies in patients with chronic CAD and/or PAD and diabetes, including single antiplatelet therapy with ticagrelor, a more potent P2Y_12_ receptor antagonist, and dual antiplatelet therapy (DAPT; Table 2) [35–41]. In the Examining Use of Ticagrelor in Peripheral Artery Disease (EUCLID) trial, there was no significant difference in the incidence of acute limb ischaemia with ticagrelor compared with clopidogrel (1.7% of patients treated with ticagrelor vs 1.7% of patients treated with clopidogrel, HR = 1.03, 95% CI 0.79–1.33; *P* = 0.85) [39]. In contrast, the Trial to Assess the Effects of SCH 530348 in Preventing Heart Attack and Stroke in Patients With Atherosclerosis (TRA 2°P-TIMI 50) that compared vorapaxar with placebo reported mixed results [42, 43]. Vorapaxar was effective in reducing the thrombotic risk in patients with prior MI and diabetes [43], but the incidence of MACE was similar between vorapaxar and placebo in patients with PAD and diabetes (11.3% vs 11.9%; HR = 0.94, 95% CI 0.78–1.14; *P* = 0.53) [42]. DAPT was found to have beneficial effects in patients with acute coronary syndrome and diabetes [35]; however, heterogeneous results were reported for intensified antiplatelet therapies in patients with chronic CVD and diabetes [37, 39, 40]. Furthermore, The Effect of Ticagrelor on Health Outcomes in Diabetes Mellitus Patients Intervention Study (THEMIS) demonstrated a significant reduction in the incidence of a composite of CV death, MI or stroke with ticagrelor plus aspirin versus placebo plus aspirin in 19,220 patients aged ≥ 50 years with stable CAD and type 2 diabetes (7.7% vs 8.5%; HR = 0.90, 95% CI 0.81–0.99; *P* = 0.04). This was at a cost of significantly increased Thrombolysis in Myocardial Infarction major bleeding and intracranial haemorrhage (2.2% vs 1.0%; HR = 2.32, 95% CI 1.82–2.94; *P* < 0.001 and 0.7% vs 0.5%; HR = 1.71, 95% CI 1.18–2.48; *P* = 0.005, respectively) [41]. In the Clopidogrel and Aspirin for Reduction of Emboli in Symptomatic Carotid Stenosis (CARESS) study, DAPT with clopidogrel and aspirin was shown to reduce asymptomatic microembolic signals in patients with carotid stenosis by 37.3% after 7 days with no life‑threatening or major bleeding. However, the CARESS study population was small (N = 100) [44]. Therefore, there is a need for improved antithrombotic management strategies in patients with chronic CAD and/or PAD and diabetes.

**Table 2:** Outcomes of trials investigating intensified antiplatelet therapies in patients with diabetes and CAD and/or PAD

Trial	Study population	Antiplatelet therapy	Comparator	Primary endpoint	Patients with diabetes (n)	Events in patients with diabetes (vs comparator, % of patients)
CHARISMA [136]	Stable CAD with high atherothrombotic risk	Aspirin plus clopidogrel	Aspirin	CV mortality, MI or stroke at 28 months	6555	16.5% vs 16.1% with nephropathy (HR = 1.0, 95% CI 0.8–1.3)
DAPT [38]	Stable CAD or ACS treated with DES or BMS implantation	Aspirin plus clopidogrel or prasugrel	Aspirin	Stent thrombosis, death, MI or stroke at 30 months	3391	6.6 vs 7.0 (HR = 0.92, 95% CI 0.71–1.20)
EUCLID [39]	Symptomatic PAD or previous revascularization of the lower limbs	Ticagrelor	Clopidogrel	CV mortality, MI or stroke at 36 months	5345	16.2 vs 15.6 (HR = 1.11, 95% CI 0.96–1.28)^a^
PEGASUS-TIMI 54 [37]	History of MI (within the prior 3 years) and additional atherothrombotic risk factor^b^	Aspirin plus ticagrelor	Aspirin	CV mortality, MI or stroke at 36 months	6806	10.0 vs 11.6 (HR = 0.84, 95% CI 0.72–0.99)^c^
THEMIS [41]	T2DM and stable CAD receiving anti-hyperglycaemic drugs < 6 months	Aspirin plus ticagrelor	Aspirin	CV mortality, MI or stroke at 36 months	19,220	6.9 vs 7.6 (HR = 0.9, 95% CI 0.81–0.99)^a^
THEMIS-PCI [40]	T2DM and stable CAD receiving anti-hyperglycaemic drugs < 6 months and previous PCI	Aspirin plus ticagrelor	Aspirin	CV mortality, MI or stroke at 40 months	11,154	7.3 vs 8.6 (HR = 0.85, 95% CI 0.74–0.97)

ACS, acute coronary syndrome; BMS, bare-metal stent; CAD, coronary artery disease; CI, confidence interval; CV, cardiovascular; DAPT, dual antiplatelet therapy; DES, drug-eluting stent; HR, hazard ratio; KM, Kaplan–Meier; MI, myocardial infarction; PAD, peripheral artery disease; PCI, percutaneous coronary intervention; T2DM, type 2 diabetes mellitus

^a^KM% at month 36

^b^Age ≥ 65 years, diabetes requiring medication, second prior MI, chronic renal dysfunction, multivessel CAD

^c^Pooled ticagrelor doses

Recently, dual pathway approaches that combine an antiplatelet with an anticoagulant have also been investigated in patients with chronic CVD and diabetes. The Cardiovascular Outcomes for People Using Anticoagulation Strategies (COMPASS) trial, which investigated the safety and efficacy of the anticoagulant rivaroxaban at a ‘vascular dose’ [5 mg twice daily (bid) or 2.5 mg bid plus aspirin] compared with aspirin alone, demonstrated a reduced incidence of both MACE and MALE in patients with chronic CAD and/or PAD with rivaroxaban 2.5 mg bid plus aspirin treatment [45–47]. This relative risk reduction was consistent in patients with and without diabetes, along with a relative risk reduction in all-cause death and the composite of CV death, MI or stroke in both patient groups and a consistent rate of net clinical benefit outcomes (MI, stroke, CV death, fatal bleeding or symptomatic critical organ bleeding) [46, 48]. Additionally, a risk stratification analysis of the COMPASS trial demonstrated that patients with diabetes represent a subgroup that has an elevated baseline risk of MACE and may, therefore, benefit the most from treatment with rivaroxaban [49]. This is reflected in the increased absolute risk reduction for MACE in patients with diabetes and the notable threefold greater reduction in mortality compared with patients without diabetes [50]. The reduction in MACE events in the COMPASS trial was primarily driven by a 42% reduction in the risk of stroke [45, 51]. Moreover, a recent subanalysis showed that this reduction was consistent in patients with a high risk of stroke at enrolment, such as those who have previously experienced a stroke or patients with diabetes [51]. The increased risk of International Society on Thrombosis and Haemostasis major bleeding with vascular dose rivaroxaban and low-dose aspirin observed in the overall COMPASS trial was also consistent in patients with and without diabetes [45, 50].

Based on the results from the COMPASS trial, the 2019 European Society of Cardiology (ESC) guidelines for the diagnosis and management of chronic coronary syndromes and the 2019 ESC/European Association for the Study of Diabetes (EASD) guidelines for diabetes, pre-diabetes and CVD recommend that the addition of a second antithrombotic drug to aspirin should be considered in patients with a high risk of ischaemic events and without a high risk of bleeding [4, 7]. This second antithrombotic drug may be clopidogrel 75 mg once daily (od), prasugrel 10 mg od or 5 mg od for patients with a body weight < 60 kg or age > 75 years, ticagrelor 60 mg bid or rivaroxaban ‘vascular dose’ 2.5 mg bid [4, 7]. Patients with a high risk of ischaemic events include those with diffuse multivessel CAD with at least one of the following: diabetes requiring medication, recurrent MI, PAD or CKD with an estimated glomerular filtration rate (eGFR) of 15–59 mL/min/1.73 m^2^ [7].

More recently, the Vascular Outcomes Study of ASA [Acetylsalicylic Acid] Along With Rivaroxaban in Endovascular or Surgical Limb Revascularization for PAD (VOYAGER PAD) trial investigated the safety and efficacy of dual pathway inhibition rivaroxaban ‘vascular dose’ 2.5 mg bid plus aspirin compared with aspirin alone in patients with PAD who had undergone revascularization within 10 days [52]. A reduction in the incidence of the primary efficacy outcome (a composite of acute limb ischaemia, major amputation for vascular causes, MI, ischaemic stroke or CV-related mortality) was observed with treatment with rivaroxaban plus aspirin. This benefit was consistent in patients with and without diabetes. A significant increase in the primary safety outcome, Thrombolysis in Myocardial Infarction major bleeding, was not observed in the overall trial; however, the risk of this outcome was significantly increased in patients with diabetes compared with patients without diabetes. Based on these results, specific recommendation updates have been included in the European label for rivaroxaban for vascular dose rivaroxaban plus low-dose aspirin for the prevention of atherothrombotic events in patients with symptomatic PAD at high risk of ischaemic events, to include those with a recent lower-extremity revascularization or diabetes [53]. Guidelines now recommend dual pathway inhibition with rivaroxaban 2.5 mg bid plus aspirin 100 mg od in patients with symptomatic PAD undergoing peripheral revascularization and should be considered following peripheral revascularization in patients with symptomatic PAD without an increased bleeding risk [54, 55].

## Management strategies for stroke prevention in patients with diabetes and AF

The Congestive heart failure, Hypertension, Age ≥ 75 years (2 points), Diabetes mellitus, Stroke or transient ischaemic attack (2 points), Vascular disease, Age 65–74, Sex category (female) (CHA_2_DS_2_-VASc) risk score can be used to predict the risk of stroke in patients with AF. An increase in the score correlates with an increased risk of stroke, with a score ≥ 2 corresponding to a high risk of stroke [56]. Patients with a prior stroke or transient ischaemic attack or those aged ≥ 75 years are considered to be at high risk of experiencing a stroke by the score. Furthermore, patients are additionally classified as high risk by the CHA_2_DS_2_-VASc risk score if they have at least two of the following risk factors; diabetes, HF, hypertension, age of 65–74 years, female gender or vascular disease [56]. For male patients with a CHA_2_DS_2_-VASc score ≥ 1 and female patients with a CHA_2_DS_2_-VASc score ≥ 2, the 2019 ESC guidelines for the diagnosis and management of chronic coronary syndromes, the 2020 ESC guidelines for the management of AF and the 2019 American Heart Association/American College of Cardiology/Heart Rhythm Society guidelines for the management of AF recommend the use of either vitamin K antagonists (VKAs) or non-vitamin K antagonist oral anticoagulants (NOACs), with a preference for NOACs where suitable, for the prevention of thromboembolic events [7, 31, 57]. Contrastingly, Japanese guidelines use the Congestive heart failure, Hypertension, Age ≥ 75 years, Diabetes mellitus, Stroke or transient ischaemic attack (2 points) (CHADS_2_) score. Each of the four NOACs are recommended for patients with a CHADS_2_ score of ≥ 2 in the Japanese Circulation Society guidelines for AF, while apixaban and dabigatran are recommended for patients with a score of 1 and edoxaban and rivaroxaban may be considered as a result of exclusion of patients with a CHADS_2_ score of 1 in their phase III studies [58]. Canadian guidelines also use an alternative stroke risk assessment algorithm, the Canadian Cardiovascular Society algorithm or CHADS-65, in which every patient aged ≥ 65 years is recommended a NOAC, as are patients aged < 65 years with a CHADS_2_ score of ≥ 1 [59]. Therefore, based on the Canadian and Japanese guidelines, every patient with AF and concomitant diabetes should receive a NOAC [58, 59].

**Fig. 3:**
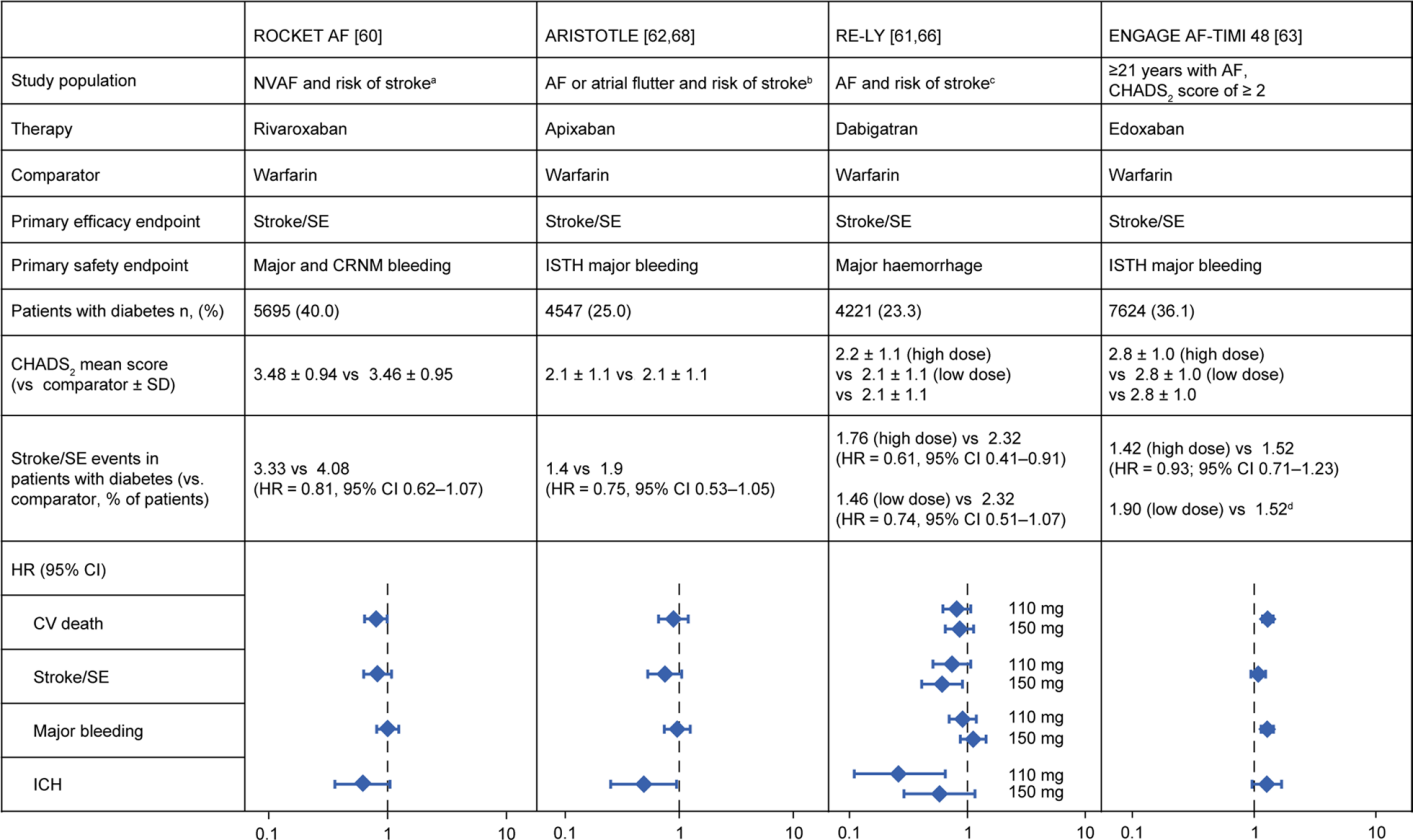
Summary of outcomes of subgroup analyses for NOACs in patients with AF and diabetes. ^a^Indicated by a history of stroke, transient ischaemic attack or SE, or ≥ 2 of the following risk factors; heart failure or a left ventricular ejection fraction of ≤ 35, hypertension, age of ≥ 75 years or diabetes (CHADS_2_ score of ≥ 2). ^b^Indicated by a history of stroke, transient ischaemic attack or SE, or systematic heart failure within prior 3 months or a left ventricular ejection fraction of ≤ 40%, hypertension requiring pharmacological treatment, age of ≥ 75 years or diabetes. ^c^Indicated by a history of stroke or transient ischaemic attack, New York Heart Association class II or higher heart failure symptoms ≤ 6 months before screening, a left ventricular ejection fraction of ≤ 40%, age of ≥ 75 years or age 65–74 years with diabetes, hypertension or coronary artery disease. ^d^HR and 95% CI not reported. AF, atrial fibrillation; CI, confidence interval; CHADS_2_, Congestive heart failure, Hypertension, Age ≥ 75 years, Diabetes mellitus, Stroke or transient ischaemic attack (2 points); CRNM, clinically relevant non-major bleeding; HR, hazard ratio; ICH, intracranial haemorrhage; ISTH, International Society on Thrombosis and Haemostasis; NOAC, non-vitamin K antagonist oral anticoagulant; NVAF, non-valvular atrial fibrillation; SD, standard deviation; SE, systemic embolism

The efficacy and safety of NOACs in the prevention of ischaemic stroke/systemic embolism (SE) in patients with AF have been demonstrated in four large phase III trials comparing NOACs with warfarin—Apixaban for Reduction in Stroke and Other Thromboembolic Events in Atrial Fibrillation (ARISTOTLE) for apixaban; Effective Anticoagulation With Factor Xa Next Generation in Atrial Fibrillation–Thrombolysis in Myocardial Infarction 48 (ENGAGE AF-TIMI 48) for edoxaban, Randomized Evaluation of Long-Term Anticoagulation Therapy (RE-LY) for dabigatran; and Rivaroxaban Once Daily Oral Direct Factor Xa Inhibition Compared with Vitamin K Antagonism for Prevention of Stroke and Embolism Trial in Atrial Fibrillation (ROCKET AF) for rivaroxaban [60–63]. A meta-analysis and subgroup analyses of these phase III trials showed that the favourable efficacy and safety profile of NOACs versus warfarin was similar in patients with and without diabetes (Fig. 3) [64–68]. A more recent meta-analysis published in 2020 of 58,634 patients from these four phase III trials highlights the same conclusions [69]. However, there are some efficacy and safety differences in the profiles of the NOACs in patients with diabetes that are worth noting.

In ROCKET AF, patients with AF were randomized to receive rivaroxaban 20 mg od or warfarin [60]. Approximately 40% of these patients had concomitant diabetes, the highest proportion of the four trials [64]. An 18% reduction in stroke/SE was observed in patients with AF and diabetes treated with rivaroxaban compared with those treated with warfarin [65]. Similar incidence rates of efficacy and safety outcomes were seen in patients with and without diabetes, although the absolute stroke risk reduction with rivaroxaban was numerically larger in those with diabetes [65]. Furthermore, rivaroxaban reduced the risk of CV mortality by 20% in patients with diabetes compared with warfarin [65]. It should be noted, however, that diabetes was an inclusion criterion associated with stroke risk in patients with AF. As a result, more patients with diabetes had a CHADS_2_ score of 5 or 6 than patients without diabetes, yet less than half of patients with diabetes had prior stroke or transient ischaemic attack and were younger on average compared with patients without diabetes [65]. Greater risk reductions, therefore, may be expected in the diabetes subgroup versus the subgroup without diabetes. Nevertheless, the similar rates of the efficacy and safety outcomes suggest that diabetes may confer a substantial risk of stroke; a hypothesis confirmed with 2-year modelling of event rates for patients with diabetes using the co-morbid profiles of patients without diabetes [65].

In ARISTOTLE, one-quarter of patients had AF and concomitant diabetes [62, 64]. Apixaban was superior to warfarin with respect to the primary efficacy endpoint of stroke/SE in patients with diabetes (HR = 0.75, 95% CI 0.53–1.05). In the overall ARISTOTLE population, rates of the primary safety outcome (International Society of Thrombosis and Haemostasis major bleeding) were lower with apixaban compared with warfarin; however, this observation was not seen in patients with diabetes where the rates of major bleeding were similar between apixaban- and warfarin-treated groups [68]. In contrast, the rates of the primary efficacy and safety endpoints were reduced with apixaban versus warfarin in patients without diabetes [68].

RE-LY investigated the efficacy and safety of dabigatran (150 mg bid or 110 mg bid) versus warfarin in patients with AF, and 23% of these patients also had diabetes at baseline [61, 64]. Both dabigatran doses demonstrated a reduction in stroke/SE and intracranial bleeding compared with warfarin in patients with and without diabetes [66]. However, the absolute stroke risk reduction with dabigatran was greater in patients with diabetes than in those without [66].

In ENGAGE AF-TIMI 48, 36% of patients had diabetes at baseline [64]. Edoxaban 60 mg od reduced the risk of CV mortality compared with warfarin in patients with or without diabetes and displayed a similar efficacy in preventing stroke/SE [67]. Patients without diabetes, however, had a significantly lower risk of bleeding [67].

Further evidence from randomized controlled trials in patients with AF undergoing percutaneous coronary intervention have also shown differences between the NOACs in outcomes for patients with or without diabetes. In Randomized Evaluation of Dual Antithrombotic Therapy With Dabigatran versus Triple Therapy With Warfarin in Patients With Nonvalvular Atrial Fibrillation Undergoing Percutaneous Coronary Intervention (RE-DUAL PCI), the incidence of the primary efficacy endpoint, a composite of time to death, first thromboembolic event (stroke/SE or MI) or unplanned revascularization, was similar between the dabigatran dual therapy (dabigatran 110 mg or 150 mg bid and clopidogrel or ticagrelor) and warfarin triple therapy (warfarin, clopidogrel or ticagrelor, and aspirin) treatment arms for patients with and without diabetes [70]. In contrast, the An Open-Label, 2 × 2 Factorial, Randomized Controlled, Clinical Trial to Evaluate the Safety of Apixaban Versus Vitamin K Antagonist and Aspirin Versus Aspirin Placebo in Patients With Atrial Fibrillation and Acute Coronary Syndrome or Percutaneous Coronary Intervention (AUGUSTUS) trial showed that apixaban decreased the time to hospitalization or death in patients without diabetes compared with warfarin; however, this outcome was not observed in patients with diabetes [71].

The benefits of NOACs in patients with AF and diabetes have also been demonstrated by real-world evidence (RWE) [72–75]. Among patients with AF and diabetes, the risk of MACE, MALE and major bleeding, including major GI bleeding, was reduced with NOAC use compared with warfarin use in a Taiwanese retrospective cohort study [76]. In a retrospective MarketScan data analysis, treatment with rivaroxaban was not only associated with a lower risk of MACE in patients with AF and diabetes compared with treatment with warfarin, but also, a reduced risk of MALE [74]. Furthermore, a recent US electronic health record analysis, A Study Using Electronic Health Information to Learn About Rivaroxaban Compared to Warfarin in Participants With Non-valvular Atrial Fibrillation (NVAF) and Diabetes (RIVA-DM), investigated a large population of patients with nonvalvular AF and type 2 diabetes [77]. Not only did RIVA-DM demonstrate significant reductions in typical CV endpoints such as stroke/SE, CV death, critical organ bleeding and intracranial haemorrhage with rivaroxaban versus warfarin, significant reductions in clinically relevant outcomes to patients with diabetes were also observed, including kidney, limb and ophthalmic complications [77, 78]. Reductions in CV and limb events were also seen with dual pathway inhibition with rivaroxaban plus aspirin versus aspirin alone in patients with PAD and concomitant diabetes who had undergone revascularization within 10 days in VOYAGER PAD and in patients with PAD or CAD and concomitant diabetes in COMPASS [50, 52]. This is important considering the significant risk of lower limb amputation in patients with diabetes [74].

Additionally, a Taiwanese nationwide retrospective cohort study of 4930 patients with AF demonstrated that patients treated with NOACs aged < 65 years or with a medication possession ratio of ≥ 80% (inferring adherence) were significantly less likely to develop new-onset diabetes compared with patients receiving warfarin [79] Therefore, adherence to NOACs may not only provide beneficial effects on CV and limb outcomes in patients with AF and diabetes, but may also reduce the risk of patients with AF developing diabetes.

## Considering renal impairment in thromboembolic prevention strategies for patients with AF and diabetes

Hyperglycaemia can result in macrovascular complications in patients with diabetes, including stroke, and microvascular complications, including diabetic nephropathy [80]. Patients with AF and/or diabetes have an increased risk of renal impairment, with diabetes being the most common cause of CKD worldwide [81, 82]. The presence of CKD in the natural history of patients with diabetes predisposes them to AF [83]. At the same time, CKD is a risk factor for AF [84]. Worsening of renal function over time is commonly observed in patients with AF treated with anticoagulants [85–88] and has been shown to be amplified in patients with concomitant diabetes [85, 86]. Furthermore, it is estimated that 30–40% of patients with diabetes develop diabetic kidney disease [82]. The progression of CKD increases the risk of CV death in patients with and without diabetes [89–91]. The ESC/EASD 2019 guidelines have identified CKD and microalbuminuria in diabetes as markers of high CV risk, with microalbuminuria also being a marker for the development of renal dysfunction [4].

CKD is associated with an increased risk of thromboembolic events and bleeding in patients with AF [92, 93]. It is, therefore, important to evaluate kidney function when managing patients with AF and diabetes, especially when choosing the type and dose of anticoagulant [4, 94]. Due to the partial elimination of all four NOACs via the kidneys, dose reductions are necessary to avoid drug accumulation in patients with renal impairment, in line with label recommendations [94]. Guidelines recommend assessing renal function in anticoagulated patients with AF at least yearly and proportionately more often in patients with impaired renal function to detect changes in kidney function and adjust the anticoagulant dose accordingly [30, 94].

Patients with AF treated with anticoagulants may be at risk of renal function decline due to anticoagulant-related nephropathy (ARN), which is a form of acute kidney injury [95]. ARN can result from excessive anticoagulation and is most commonly associated with warfarin. However, NOACs have also been reported to be associated with ARN—most frequently dabigatran [95–97]. Diabetes and CKD have been identified as risk factors for the development of ARN [97]. A recent meta-analysis has shown that patients with ARN are at significantly greater risk of mortality at 5 years compared with patients without ARN receiving anticoagulation (HR = 1.91, 95% CI 1.22–3.00) [97]. ARN is also associated with increased renal morbidity and accelerated progression of CKD [96, 98], further complicating the management of patients with AF and concomitant diabetes and renal impairment.

Patients with CKD have reduced levels of regulatory molecules or abnormal regulatory molecules that act to inhibit calcium deposition in non-osseous tissue and promote the incorporation of calcium into bone [99]. In addition, VKAs prevent the activation of the matrix Gla protein, thereby promoting vascular calcification in the kidneys (renovascular calcification) [100] and elsewhere in patients with CKD (for example, peripheral arteries, heart valves and coronary arteries) [101, 102]; this causes a further decline of renal function and increased vascular morbidity and mortality [99, 103]. In contrast, RWE studies have demonstrated a beneficial effect of NOACs, in particular rivaroxaban and dabigatran, in reducing the risk of renal function decline due to anticoagulation compared with VKAs in patients with AF [75, 88, 104, 105]. This effect has also been frequently observed in patients with co-morbid diabetes [75, 106]. This may be attributed to the activation of prothrombin by factor Xa; factor Xa interacts with the protease-activated receptors 1 and 2, therefore suggesting that factor Xa inhibitors prevent thrombin-mediated effects such as inflammation, tissue fibrosis and vascular remodelling [107].

Due to the renal excretion of NOACs and an increased risk of bleeding in patients with kidney dysfunction, it is important that the appropriate NOAC dose is administered [108]. However, on the basis of more recent evidence, continuing with a NOAC in patients with impaired kidney function rather than switching to a VKA could result in better patient outcomes [75, 88, 104–106]. Apixaban, edoxaban and rivaroxaban are contraindicated in patients with a creatinine clearance (CrCl) < 15 mL/min while dabigatran (at a lowest dose of 110 mg bid) is contraindicated in patients with a CrCl < 30 mL/min [53, 109–111]. Additionally, a reduced dose of dabigatran may be considered for patients at high risk of bleeding, while a reduced dose of edoxaban may be considered in patients with a CrCl 15–50 mL/min, body weight ≤ 60 kg or those receiving treatment with P-glycoprotein inhibitors [110, 111]. A reduced dose of apixaban is also required for patients with AF and at least two of three risk factors: serum creatinine ≥ 1.5 mg/dL, aged ≥ 80 years or with a body weight ≤ 60 kg [109]. Thus, by prescribing the appropriate type of anticoagulant to patients with diabetes, in addition to using a dose appropriate to their clinical characteristics, it might be possible to preserve their renal function and prevent adverse limb events while protecting them from stroke and fatal CV events. The importance of reducing renal function decline and the potential impact of the choice of anticoagulant on renal outcomes in patients with AF have also been highlighted in the 2019 update to the American College of Cardiology/American Heart Association/Heart Rhythm Society guidelines on the management of AF, stating that *‘over time, NOACs (particularly dabigatran and rivaroxaban) may be associated with lower risks of adverse renal outcomes than warfarin in patients with AF’* [31]. This is particularly relevant in patients with AF and diabetes given the impact of worsening GFR on mortality and CV death [31, 112, 113]. Thus, in the context of comprehensive preventive strategies in this very vulnerable population, warfarin is not the preferred anticoagulant at an early stage in patients with diabetes, AF or in the early stages of diabetic renal disease.

## The role of anti-hyperglycaemic agents in the management of CV risk in patients with diabetes

GLP-1 is an incretin hormone released from the gut in response to glucose, upregulating insulin secretion and downregulating glucagon release. While the intrinsic GLP-1 peptide has a short half-life, synthetic GLP-1 receptor agonists typically have longer half-lives and bind with similar affinity to the GLP-1 receptor, thereby acting to prevent hyper- and hypo-glycaemia in patients with diabetes [114]. In contrast, SGLT2 inhibitors inhibit SGLT2 activity, a low-affinity sodium-glucose co-transporter in the proximal tubule of the nephron, reducing glucose reabsorption and blood glucose levels in patients with diabetes [115].

By regulating blood glucose levels, anti-glycaemic agents such as GLP-1 receptor agonists and SGLT2 inhibitors aim to prevent hyperglycaemia and resultant vascular complications in patients with diabetes [80]. Several CV outcome trials have indicated that GLP-1 receptor agonists and SGLT2 inhibitors have CV benefit in patients with CVD or at high CV risk [4, 116]. A meta-analysis of eight CV outcome trials in patients with type 2 diabetes showed a significant reduction in non-fatal stroke with GLP-1 receptor agonists versus placebo [117]. In contrast, the EMPA-REG trial did not show a reduction in the risk of non-fatal stroke with the SGLT2 inhibitor empagliflozin versus placebo in patients with type 2 diabetes and established CVD [118]. These studies were not in the setting of AF [117, 118]. Both GLP-1 receptor agonists and SGLT2 inhibitors have been shown to reduce the risk of MACE, but some SGLT2 inhibitors reduced the risk of MACE only in patients with prior MI [117–119].

Clinical trial data also suggest a renoprotective effect of both GLP-1 receptor agonists and SGLT2 inhibitors in patients with diabetes, which may contribute to their CV benefits [120–123]. Other evidence has shown a reduction in limb events with GLP-1 receptor agonists. For example, liraglutide reduced the risk of amputations associated with diabetes-related foot ulcerations compared with placebo in patients with type 2 diabetes and a high risk of CV events [124]. The incidence of major adverse limb events was also lower in patients with type 2 diabetes receiving GLP-1 receptor agonists compared with those receiving DPP4 inhibitors [125].

As a result of these CV benefits, the 2022 American Diabetes Association (ADA) standards of medical care in diabetes clinical practice recommendations advise that an SGLT2 inhibitor or GLP-1 receptor agonist with demonstrated CVD benefit should be given as part of a regimen in patients with type 2 diabetes with established atherosclerotic CVD. This recommendation also applies to patients with indicators of high CV risk, established kidney disease or HF, with consideration for patient-specific factors, and is independent of glycated haemoglobin [126]. In patients with type 2 diabetes and established atherosclerotic CVD or multiple CV risk factors, an SGLT2 inhibitor or GLP-1 receptor agonist with demonstrated CV benefit is recommended to reduce the risk of MACE, whereas SGLT2 inhibitors are further recommended in these patients to reduce the risk of hospitalization for HF and in those with diabetic kidney disease [127].

The ADA and EASD 2019 consensus report recommends SGLT2 inhibitors for the reduction of MACE, CV mortality and hospitalization for HF in patients with diabetes and concomitant HF or CKD [128]. SGLT2 inhibitors are recommended to reduce the risk of hospitalization for HF in patients with diabetes in the 2019 ESC/EASD guidelines for diabetes, pre-diabetes and CVD, and GLP-1 receptor agonists may be considered for the treatment of diabetes in patients with HF. Moreover, these guidelines recommend GLP-1 receptor agonists and SGLT2 inhibitors as first-line glucose-lowering treatments in patients with type 2 diabetes and CVD, or at high to very high CV risk, to reduce CV events and mortality [4]. It should also be noted that SGLT2 inhibitors are recommended in patients with an eGFR of 30 to < 90 mL/min/1.73 m^2^, whereas GLP-1 receptor agonists should be considered in patients with an eGFR > 30 mL/min/1.73 m^2^ [43].

Glucose-lowering diabetic medications have been shown to reduce the risk of AF development, consequently reducing the risk of stroke [24]. It is hypothesized that this is achieved through the antioxidant and anti-inflammatory effects of the diabetic medications, thereby acting on the pathophysiology of both diabetes and AF (Fig. 2) [129, 130]. An analysis of the Dapagliflozin Effect on Cardiovascular Events–Thrombolysis in Myocardial Infarction 58 (DECLARE-TIMI 58) trial with the SGLT2 inhibitor dapagliflozin showed a reduction of new and recurrent AF and atrial flutter events [131]. Large cohort studies have shown that metformin and thiazolidinediones are associated with a reduction in the risk of new-onset AF [129, 130], and a recent meta-analysis demonstrated that treatment with thiazolidinediones reduced the risk of developing AF by 27% [132]. Glycaemic fluctuations, however, have been shown to correlate with increased oxidative stress compared with hyperglycaemia, and, therefore, may have a greater contribution to the initiation of CV complications and AF in diabetes [133, 134]. Moreover, long-term glycaemic variability was significantly associated with new-onset AF in a recent cohort study; multiple Cox regression demonstrated that higher glycated haemoglobin levels were a predictor of new-onset AF following adjustment for age, body mass index, left ventricular mass index or left atrium diameter or if the variability of levels was determined by standard deviation or coefficient of variation [135]. This suggests that the management of diabetes should be focused on limiting glycaemic fluctuations in addition to decreasing blood glucose levels [24].

## Conclusion

Progress has been made in the development of pharmacological therapies for the prevention of CV events in patients in sinus rhythm with diabetes and CAD and/or PAD, as well as in patients with diabetes and AF. Randomized controlled trial data suggest a benefit for rivaroxaban, as part of a dual pathway approach with aspirin, in the reduction of MACE and MALE in patients with sinus rhythm with diabetes and CAD and/or PAD. Evidence of the benefits of NOACs for stroke prevention in patients with diabetes and AF is accumulating, with data from clinical trials now being supported with emerging RWE. The impact of renal function decline on CV outcomes in patients with AF has been increasingly recognized, which is particularly important for patients with diabetes who have a high risk of developing kidney disease. Future management approaches for patients with diabetes who are at an increased risk of CV events should consider aspects such as renal and lower limb function, in addition to the prevention of CV events. Such optimized care would not only protect the patient from CV events but would also reduce their risk of lower limb amputation and dialysis; complications that are particularly concerning for patients with diabetes.

## Data Availability

Not applicable.

## Abbreviations

ADA: American Diabetes Association
AF: atrial fibrillation
ARISTOTLE: Apixaban for Reduction in Stroke and Other Thromboembolic Events in Atrial Fibrillation
ARN: anticoagulant-related nephropathy
ASCEND: A Study of Cardiovascular Events in Diabetes
APPRAISE: Aspirin in Primary Prevention of Cardiovascular Disease in Diabetes
ASPREE: Aspirin in Reducing Events in the Elderly
AUGUSTUS: An Open-Label, 2 × 2 Factorial, Randomized Controlled, Clinical Trial to Evaluate the Safety of Apixaban Versus Vitamin K Antagonist and Aspirin Versus Aspirin Placebo in Patients With Atrial Fibrillation and Acute Coronary Syndrome or Percutaneous Coronary Intervention
bid: twice daily
CAD: Coronary artery disease
CAPRIE: Clopidogrel Versus Aspirin in Patients at Risk of Ischaemic Events
CARESS: Clopidogrel and Aspirin for Reduction of Emboli in Symptomatic Carotid Stenosis
CHA_2_DS_2_-VASc: Congestive heart failure, Hypertension, Age ≥ 75 years (2 points), Diabetes mellitus, Stroke or transient ischaemic attack (2 points), Vascular disease, age 65–74, Sex category (female)
CHADS_2_: Congestive heart failure, Hypertension, Age ≥ 75 years, Diabetes mellitus, Stroke or transient ischaemic attack (2 points) score
CHARISMA: Clopidogrel for High Atherothrombotic Risk and Ischemic Stabilization, Management, and Avoidance
CI: confidence interval
CKD: chronic kidney disease
COMPASS: Cardiovascular Outcomes for People Using Anticoagulation Strategies
CrCl: creatinine clearance
CV: cardiovascular
CVD: cardiovascular disease
DAPT: dual antiplatelet therapy
DECLARE-TIMI 58: Dapagliflozin Effect on Cardiovascular Events–Thrombolysis in Myocardial Infarction 58
EASD: European Association for the Study of Diabetes
eGFR: estimated glomerular filtration rate
ESC: European Society of Cardiology
ENGAGE AF-TIMI 48: Effective Anticoagulation With Factor Xa Next Generation in Atrial Fibrillation–Thrombolysis in Myocardial Infarction 48
EUCLID: Examining Use of Ticagrelor in Peripheral Artery Disease
GI: gastrointestinal
GLP-1: glucagon-like peptide-1
GUSTO: Global Use of Strategies to Open Occluded Coronary Arteries
HF: heart failure
HR: hazard ratio
MACE: major adverse cardiovascular events
MALE: major adverse limb events
MI: myocardial infarction
NOAC: non-vitamin K antagonist oral anticoagulant
od: once daily
PAD: peripheral artery disease
RE-DUAL PCI: Randomized Evaluation of Dual Antithrombotic Therapy With Dabigatran Versus Triple Therapy With Warfarin in Patients with Nonvalvular Atrial Fibrillation Undergoing Percutaneous Coronary Intervention
RE-LY: Randomized Evaluation of Long-Term Anticoagulation Therapy
REACH: REduction of Atherothrombosis for Continued Health
RIVA-DM: A Study Using Electronic Health Information to Learn About Rivaroxaban Compared to Warfarin in Participants With Non-valvular Atrial Fibrillation (NVAF) and Diabetes
ROCKET AF: Rivaroxaban Once Daily Oral Direct Factor Xa Inhibition Compared With Vitamin K Antagonism for Prevention of Stroke and Embolism Trial in Atrial Fibrillation
RWE: real-world evidence
SE: systemic embolism
SGLT2: sodium-glucose co-transporter-2
THEMIS: The Effect of Ticagrelor on Health Outcomes in Diabetes Mellitus Patients Intervention Study
TRA 2°P-TIMI 50: Trial to Assess the Effects of SCH 530348 in Preventing Heart Attack and Stroke in Patients With Atherosclerosis
VKA: vitamin K antagonist
VOYAGER PAD: Vascular Outcomes Study of ASA [acetylsalicylic acid] Along with Rivaroxaban in Endovascular or Surgical Limb Revascularization for PAD
